# Predictors of Cognitive Decline in Healthy Middle-Aged Individuals with Asymptomatic Alzheimer’s Disease

**DOI:** 10.21203/rs.3.rs-2577025/v1

**Published:** 2023-02-28

**Authors:** Raghav Tandon, Liping Zhao, Caroline M. Watson, Morgan Elmor, Craig Heilman, Katherine Sanders, Chadwick M. Hales, Huiying Yang, David W. Loring, Felicia C. Goldstein, John J. Hanfelt, Duc M. Duong, Erik C.B. Johnson, Alzheimer’s Disease Neuroimaging Initiative, Aliza P. Wingo, Thomas S. Wingo, Blaine R. Roberts, Nicholas T. Seyfried, Allan I. Levey, Cassie S. Mitchell, James J. Lah

**Affiliations:** 1. Department of Biomedical Engineering, Georgia Institute of Technology; 2. Center for Machine Learning, Georgia Institute of Technology; 3. Department of Biostatistics and Bioinformatics, Emory School of Public Health; 4. Emory Goizueta Alzheimer’s Disease Research Center; 5. Department of Neurology, Emory School of Medicine; 6. Center for Neurodegenerative Disease, Emory University; 7. Department of Biochemistry, Emory School of Medicine; 8. Department of Psychiatry, Emory School of Medicine; 9. Division of Mental Health, Atlanta VA Medical Center, GA, USA

## Abstract

Alzheimer’s disease (AD) progresses through a lengthy asymptomatic period during which pathological changes accumulate prior to development of clinical symptoms. As disease-modifying treatments are developed, tools to stratify risk of clinical disease will be required to guide their use. In this study, we examine the relationship of AD biomarkers in healthy middle-aged individuals to health history, family history, and neuropsychological measures and identify cerebrospinal fluid (CSF) biomarkers to stratify risk of progression from asymptomatic to symptomatic AD. CSF from cognitively normal (CN) individuals (N=1149) in the Emory Healthy Brain Study were assayed for Aβ_42_, total Tau (tTau), and phospho181-Tau (pTau), and a subset of 134 cognitively normal, but biomarker-positive, individuals were identified with asymptomatic AD (AsymAD) based on a locally-determined cutoff value for ratio of tTau to Aβ_42_. These AsymAD cases were matched for demographic features with 134 biomarker-negative controls (CN/BM-) and compared for differences in medical comorbidities and family history. Dyslipidemia emerged as a distinguishing feature between AsymAD and CN/BM-groups with significant association with personal and family history of dyslipidemia. A weaker relationship was seen with diabetes, but there was no association with hypertension. Examination of the full cohort by median regression revealed a significant relationship of CSF Aβ_42_ (but not tTau or pTau) with dyslipidemia and diabetes. On neuropsychological tests, CSF Aβ_42_ was not correlated with performance on any measures, but tTau and pTau were strongly correlated with visuospatial perception and visual episodic memory. In addition to traditional CSF AD biomarkers, a panel of AD biomarker peptides derived from integrating brain and CSF proteomes were evaluated using machine learning strategies to identify a set of 8 peptides that accurately classified CN/BM- and symptomatic AD CSF samples with AUC of 0.982. Using these 8 peptides in a low dimensional t-distributed Stochastic Neighbor Embedding analysis and k-Nearest Neighbor (k=5) algorithm, AsymAD cases were stratified into “Control-like” and “AD-like” subgroups based on their proximity to CN/BM- or AD CSF profiles. Independent analysis of these cases using a Joint Mutual Information algorithm selected a set of 5 peptides with 81% accuracy in stratifying cases into AD-like and Control-like subgroups. Performance of both sets of peptides was evaluated and validated in an independent data set from the Alzheimer’s Disease Neuroimaging Initiative. Based on our findings, we conclude that there is an important role of lipid metabolism in asymptomatic stages of AD. Visuospatial perception and visual episodic memory may be more sensitive than language-based abilities to earliest stages of cognitive decline in AD. Finally, candidate CSF peptides show promise as next generation biomarkers for predicting progression from asymptomatic to symptomatic stages of AD.

## 1. Introduction

Pathophysiological changes of Alzheimer’s disease (AD) begin many years before the functional or cognitive decline associated with disease. For example, in individuals with dominantly inherited AD, cerebrospinal fluid (CSF) Tau begins to increase 15 years and Aβ_42_ begins to decline over 20 years prior to symptom onset^1,2^. Until a recent report of lecanemab^3^, clinical trials of anti-amyloid monoclonal antibodies^4-6^, secretase inhibitors^7,8^, and anti-tau monoclonal antibodies^9,10^ have had limited success in symptomatic AD patients. Given the long evolution of these pathologies before clinical symptoms, identifying and treating at-risk individuals during asymptomatic stages may be a more effective strategy to delay or prevent dementia onset^11^. Thus, a key to successful implementation of secondary prevention trials may lie in the ability to identify those at greatest risk for AD prior to symptom onset. It is also important to recognize that many cognitively normal (CN) individuals may have evidence of AD neuropathology at death^12,13^. Among CN controls in the National Alzheimer’s Coordinating Center (NACC) database (May 2022), 226/787 (28.7%) had moderate or frequent amyloid plaques (CERAD ≥2), 386/787 (49.2%) had neocortical neurofibrillary tangles (Braak ≥3), and 163/787 (20.7%) had both CERAD ≥2 and Braak ≥3. Therefore, simply identifying the presence of AD pathology does not imply a need for intervention. For effective deployment of preventative therapies, it is imperative to both identify the presence of silent pathology and determine those at greatest risk of developing symptomatic disease. As AD is a multifactorial neurodegenerative disorder with numerous etiopathogenic mechanisms, a number of factors may influence early disease evolution, including genetics, lifetime exposures, and medical comorbidities. Additionally, AD typically manifests as mixed pathologies which evolve and change over time^14-16^, and multiple biomarkers are likely to be required to predict underlying pathology, disease stage, and risk of clinical progression. Recent application of systems biology approaches has led to development of proteomics-based CSF biomarker panels that link to diverse brain pathologies and may refine disease-staging and support tailored therapeutic strategies^17-21^. This early work indicates that these additional biomarkers may have the ability to stratify risk of clinically symptomatic AD.

To better understand the evolution of AD in its earliest stages, we explored CSF characteristics in patients with symptomatic AD and a cohort of 1149 CN middle-aged individuals (50-75 years) in the Emory Healthy Brain Study (EHBS)^22^, including a subset of 134 individuals with CSF levels of Aβ_42_, total Tau (tTau), and phospho181-Tau (pTau) indicative of underlying AD pathology based on a locally-determined cutoff ratio of tTau to Aβ_42_. We compared this group of asymptomatic AD (AsymAD) individuals and a demographically matched group of CN and CSF biomarker-negative (CN/BM-) controls and examined the relationship of CSF AD biomarkers to comorbidities, family history, and performance on cognitive measures. Regression analyses in the full cohort of EHBS participants were performed to look at the correlation of CSF Aβ_42_, tTau, and pTau with comorbidities, family history, and neuropsychological measures. Machine learning algorithms were used to identify a set of CSF peptides that effectively discriminate CN/BM- controls from symptomatic AD cases and another set of peptides that sub-categorized AsymAD individuals into “AD-like” and “Control-like” groups. Our results reveal an association of specific risk factors and cognitive changes with asymptomatic stages of AD and identify a set of CSF biomarkers that may serve to stratify risk of conversion from asymptomatic to symptomatic stages of AD.

## 2. Methods

### 2.1 Emory Healthy Brain Study

The EHBS^22^ is a longitudinal cohort study of cognitively normal adults (50-75 years) established in 2016. It is a research study specifically focused on discovering biomarkers that predict AD and other dementias. EHBS participants are self-reported cognitively and functionally intact and free of pre-existing diagnosis of mild cognitive impairment (MCI) or any dementia. All participants complete biennial study visits which include neuropsychological testing, cardiovascular measures, brain imaging, and biospecimen collection (blood, CSF). A total of 1149 EHBS participants who had completed baseline visits and CSF collection through August 2021 were included in the current report. From this cohort, we identified 134 cognitively normal, biomarker-positive individuals with AsymAD based on measurements of Aβ_42_, tTau, and pTau using a locally defined cutoff value for tTaû42 ratio (>0.24) identified by Gaussian mixture models^23^. These individuals were matched for age, sex, and race with 134 biomarker-negative CN/BM- controls and 134 biomarker-confirmed symptomatic AD patients seen in the Emory Cognitive Neurology Clinic. AsymAD and CN/BM- controls were additionally matched for education. All individuals included in our analyses provided informed consent to participate in research protocols approved by the Emory University Institutional Review Board. Table 1 shows the descriptive statistics for the matched groups of clinical AD, AsymAD, and CN/BM- control groups (N=134 each). Statistical differences between the AsymAD and CN/BM- groups were evaluated using the McNemar-Bowker’s test for categorical variables and by paired t-test or Wilcoxon signed rank test for continuous variables depending on the distribution.

### 2.2 Neuropsychological assessment and analysis

Neuropsychological measures collected in the EHBS cohort include the Montreal Cognitive Assessment (MoCA)^24^, Number Span forward and backward^25^, Trail Making Test A&B^26^, Multilingual Naming Test (MINT)^27^, Phonemic Fluency test^28^, Category Fluency test^29^, Rey-Osterreith Complex Figure Test (RCFT)^30^, Rey Auditory Verbal Learning Test (AVLT)^31^, and Judgment of Line Orientation (JoLO)^32^. Neuropsychological test scores were not normally distributed and therefore compared using the Wilcoxon signed rank test between matched AsymAD and CN/BM- groups. Because the residual distributions of neuropsychological assessments were highly skewed and medians were better measures of central tendency, neuropsychological assessments for the entire cohort were regressed upon standardized CSF values using non-parametric median regression, while adjusting for age, sex, race and education. Median regression is a better alternative to ordinary least-squares regression because it is more robust to outliers and makes no assumptions about the distribution of the residuals. This resulted in estimation of median of the cognitive scores conditional on CSF analytes, adjusted for sociodemographics in the EHBS population. Due to sparse data, Number Span Forward, Number Span Backward and MINT were dichotomized by its median and analyzed using logistic regression to assess their relationship with the standardized CSF values, controlling for sociodemographics. To correct for multiple testing, an FDR correction was applied by the Benjamini-Hochberg method (FDR<20%). Goodness of fit statistics “R1” was calculated to assess model fit. Statistical analyses were performed using SAS^®^ 9.4 (SAS Institute Inc) or R software (www.R-proiect.org).

### 2.3 CSF collection and analysis

CSF samples from all participants were collected in a standardized fashion applying common preanalytical methods. EHBS participants were asked to fast for at least 6 hours prior to study visits. Patients donating CSF samples in the course of clinical evaluations were asked to fast prior to their lumbar puncture (LP) procedure, but failure to do so did not preclude LP and CSF collection. Most, but not all procedures, were conducted before noon. All clinicians performing LPs in the Cognitive Neurology Clinic are also active investigators in the EHBS and apply shared standard work in both settings. LPs are performed using a 24g atraumatic Sprotte spinal needle (Pajunk Medical Systems, Norcross, GA) with aspiration and, after clearing any blood contamination, CSF is transferred from syringe to 15 ml polypropylene tubes (Corning, Glendale, AZ), which are inverted several times. The CSF is aliquoted without further handling into 0.5 ml volume in 0.9 ml FluidX tubes (Azenta, Chemsford, MA) and placed into dry ice/methanol bath prior to transfer to −80°C freezers. Time from initial collection to storage at −80°C is less than 60 minutes. Aβ_42_, tTau, and pTau assays were performed on CSF samples following a single freeze-thaw cycle on a Roche Cobas e601 analyzer using the Elecsys assay platform^33^. All assays were performed in a single laboratory in the Emory Goizueta Alzheimer’s Clinical Research Unit following manufacturer’s recommended protocols. Due to skewness and outliers, median regression was also utilized to assess the effect of personal or family history of health conditions on CSF biomarkers with adjustment of age, sex, race and education. The CSF analytes were standardized to facilitate comparability of the relative importance in all regression analyses. When the CSF analytes are dependent variables, the β coefficients represent standard deviation (SD) changes of CSF level per 1-unit change in the independent variables; when CSF biomarkers are independent variables, the β coefficients indicate changes in the outcomes per one SD change in the CSF measurements. A more positive standardized β coefficient suggested a stronger association while a more negative standardized β coefficient showed a stronger inverse association.

### 2.4 Stratification of asymptomatic AD cases

#### 2.4.1 Peptide selection to discriminate healthy controls and AD cases

We recently reported CSF protein changes associated with AD from integrated discovery proteomics of brain and CSF^34^. In our current analyses, we examined expression of 75 peptides mapped to 58 unique proteins quantified by selected reaction monitoring mass spectrometry methods as detailed elsewhere^21^. To identify peptides differentiating CN/BM- controls from AD patients, a machine learning strategy of backward selection was employed using 80% of all CN/BM- and AD individuals. A linear classifier, Support Vector Machine (SVM), first used all available peptides to distinguish AD cases from CN/BM- controls. We then applied Recursive Feature Elimination (RFE)^35^ to eliminate the weakest peptides in a stepwise fashion to arrive at a smaller subset deemed important for the classification task. Recent work has shown that RFE-based biomarker selection outperforms other biomarker selection methods from proteomic datasets in supervised settings^36^. The size of the subset at which to stop the recursive process is a user defined parameter (set to 14). The choice of the classifier model has some influence on selection of peptides as the set of peptides resulting from RFE is not invariant to the choice of the classifier model. To address this, we recombined RFE with a different linear classifier (logistic regression) resulting in a second peptide set, deemed useful for classifying CN/BM- controls and AD cases. The final set of selected peptides is the intersection of these two sets and provides a more compact set of peptides which are useful for classification. A schematic of the peptide selection process is shown in Figure 2a. Eighty percent of data (CN/BM- and AD cases) were used to identify peptides, and 8 peptides were chosen and validated on the held-out set (remaining 20% data). This held-out data set played no role in peptide identification or classifier training. These peptides were also tested in a permutation test setting where the performance of the chosen peptides was compared to the performance of 100,000 randomly chosen peptide sets of the same size (n=8). Correlation analyses between all measured peptides and MoCA score was performed using Kendall-Tau correlation, which assesses the strength of monotonic association between the peptide and MoCA. MoCA scores were available for all 3 groups (i.e., CN/BM-, AsymAD, and AD). The correlation coefficients are sorted in a descending order and peptides which were chosen to discriminate controls from AD are shown in bold (Fig. 2h).

#### 2.4.2 Stratifying AsymAD cases

To determine if the 8 peptides chosen to discriminate between CN/BM- controls and AD can be used to classify AsymAD with more resolution, we used a low-dimensional representation to stratify AsymAD cases. This involved two successive steps of dimensionality reduction. The first was through the peptide selection (going from 75 to 8 peptides), and the second step used the t-distributed Stochastic Neighbor Embedding (t-SNE) algorithm to reduce these 8 peptides into 2 features. This analysis enables 2-dimensional visualization of how high-dimensional peptide data varies across different subjects. Lastly, the AsymAD cases were categorized as “Control-like” or “AD-like”, depending on which class (CN/BM- or AD) shares greater proximity with a given AsymAD case. This proximity is calculated using the K-Nearest Neighbor (KNN) algorithm (k=5). An AsymAD case is called “Control-like” if the majority of its 5 nearest neighbors are CN/BM- and “AD- like” otherwise. This is shown in Figure 3b-3e. The *APOE* genotypes of the resulting AsymAD sub-categories were analyzed for differences using the Fisher’s exact test (Fig. 3g). Peptides which are able to differentiate between these sub-categories were evaluated using an information theoretic algorithm, Joint Mutual Information (JMI)^37^, to provide an independent view for comparing sub-categories (Fig. 3h-3i).

#### 2.4.3 Evaluation of peptides in Alzheimer’s Disease Neuroimaging Initiative (ADNI)

To assess the ability of peptides identified in the EHBS cohort to discriminate between CSF from CN/BM- controls and AD and to sub-categorize individuals with AsymAD, these peptide panels were evaluated in the Alzheimer’s Disease Neuroimaging Initiative (ADNI) cohort (adni.loni.usc.edu). The ADNI was launched in 2003 as a public-private partnership, led by Principal Investigator Michael W. Weiner, MD. The primary goal of ADNI has been to test whether serial magnetic resonance imaging (MRI), positron emission tomography (PET), other biological markers, and clinical and neuropsychological assessment can be combined to measure the progression of mild cognitive impairment (MCI) and early AD. We recently performed targeted proteomics on 706 baseline CSF samples to quantify the same set of target proteins evaluated in the EHBS cohort. Baseline amyloid PET was used to ascertain presence or absence of underlying AD pathology with CN individuals with positive amyloid PET identified as AsymAD, and hypometabolism on baseline fluorodeoxyglucose (FDG) PET was used to identify AsymAD individuals who may be closer to symptomatic disease. Standardized uptake value ratios (SUVR) for florbetapir (AV45) and FDG PET were determined by ADNI investigators as described (https://adni.loni.usc.edu/methods/pet-analysis-method/pet-analysis/). Cutoff SUVR values were determined based on Youden index in ROC analyses for AV45 (>1.226) and FDG (<1.191) using results from individuals classified as CN and Dementia at baseline ADNI visit. Individuals with baseline classification of EMCI, LMCI, or SMC were not included in the ROC plot. The 8 RFE-selected peptides were analyzed in the ADNI cohort in three groups: CN/BM- (AV45≤1.226; n=203), AsymAD (CN; AV45>1.226; n=52), and AD (Dementia or MCI; AV45>1.226; n=250). Individuals without AV45 or FDG data and individuals with Dementia or MCI with AV45 SUVR≤1.226 (n=192) were not included in the analysis. The JMI-selected panel of 5 peptides was assessed for ability to discriminate between AsymAD individuals with positive (SUVR <1.191; n=10) or negative (SUVR ≥1.191; n=42) baseline FDG PET scans.

## 3. Results

### 3.1 Relationship of dyslipidemia and diabetes to asymptomatic AD and CSF Aβ_42_

Table 1 shows characteristics of the matched set of samples consisting of individuals with symptomatic AD, AsymAD, and CN/BM- controls with 134 in each group. All groups were matched for age, sex, and race, and the CN/BM- controls and AsymAD cases were also matched for education. As expected, the AD group was substantially different from both AsymAD and CN/BM- control groups in education, MoCA score, *APOE* ε4 allele frequency, and levels of Aβ_42_, tTau, and pTau (p <0.0001 for all). All p-values listed in Table 1 are for comparisons between AsymAD and CN/BM- control groups only and show significantly higher *APOE* ε4 allele frequency, lower levels of Aβ_42_ and higher levels of tTau and pTau in AsymAD compared to CN/BM- controls (p<0.0001 for all). As medical comorbidities and family history can influence AD risk, we also compared prevalence of comorbidities and frequency of positive family history in these two groups. The prevalence of hypertension, coronary artery disease (CAD), diabetes, and dyslipidemia and frequency of family history of MCI, AD, memory loss, CAD, stroke, hypertension, diabetes, and dyslipidemia are shown for the matched AsymAD and CN/BM- groups. Comparable data were not consistently collected for the clinical AD group and could not be compared. Relative to the CN/BM- control group, the AsymAD group had significantly higher prevalence of dyslipidemia (p=0.015) and diabetes (p=0.033), and a trend for higher prevalence of hypertension (p=0.11) and CAD (p=0.10). The AsymAD group also had significantly more family history of AD (p=0.0004) and dyslipidemia (p=0.030) in first degree relatives.

The impact of comorbidities and family history on CSF AD biomarkers for the entire EHBS cohort based on median regression analysis adjusted for age, sex, race, and education is presented in Table 2 (significant effects only) and **Supplemental Table S1** (all regression results of the health conditions and CSF biomarkers). Compared to participants without dyslipidemia, diabetes, and family history of AD, participants with these conditions had significantly lower Aβ_42_ (standardized parameter estimates, −0.23, −0.33, and −0.42, respectively), higher tTau:Aβ42 ratio (0.07, 0.19, and 0.16, respectively) and higher pTau:Aβ42 ratio (0.04, 0.10, and 0.08, respectively). After FDR correction, the association of dyslipidemia and family history of AD with Aβ_42_ and tTau:Aβ_42_ remained significant. The association of diabetes with tTau:Aβ_42_ (but not Aβ_42_) remained significant after FDR correction. No significant association of dyslipidemia, diabetes, or family history of AD was seen with either tTau or pTau. These results from matched AsymAD and CN/BM- cases and the median regression analyses in the entire cohort suggest that dyslipidemia and diabetes play an important role in early evolution of AD, specifically mediated through effects on Aβ_42_ rather than tTau or pTau.

### 3.2 Neuropsychological measures of visuospatial function are correlated with CSF AD biomarkers in CN individuals

**Figure 1.**
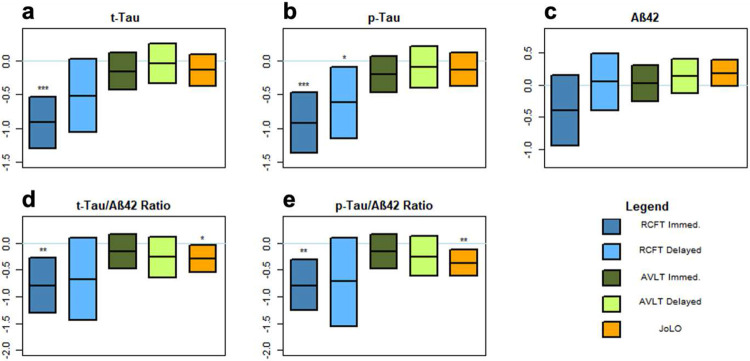
Cognitive scores regressed on CSF analytes while adjusting for age, sex, race and education. The figures show the median regression coefficients and their 95% confidence intervals for (**a**) tTau, (**b**) pTau, (**c**) Aβ_42_, (**d**) tTau:Aβ42, (**e**) pTau:Aβ_42_ in explaining 5 different neuropsychological assessments – RCFT Immediate recall, RCFT Delayed recall, AVLT Immediate recall, AVLT Delayed recall, and JoLO. The results show significant associations between tTau, pTau, tTau:Aβ_42_, and pTau:Aβ_42_ and RCFT Immediate recall, pTau and RCFT Delayed recall, tTau:Aβ_42_ and pTau:Aβ_42_ and JoLO. Aβ_42_ shows no significant association with any neuropsychologic assessment. (*p<0.05, **p<0.01, ***p<0.001)

Table 3 shows comparisons of cognitive performance in matched AsymAD and CN/BM- groups. The AsymAD group had significantly poorer performance on JoLO (p=0.0022) and tended to perform worse on RCFT delayed recall (p=0.09). No other test showed a significant difference between the AsymAD and CN/BM- groups. To further explore cognitive features that may be most sensitive to early stages of AD, we analyzed the relationship of standardized CSF AD biomarkers to neuropsychological measures in the entire EHBS cohort. A summary of significant correlations between cognitive performance and standardized CSF biomarkers are shown in Table 4, and correlations for all cognitive tests with standardized CSF analytes are provided in **Supplemental Table S2**. Levels of Aβ_42_ were not significantly correlated with any cognitive measures, but increases in tTau and pTau (as well as tTau:Aβ42 and pTau:Aβ42 ratios) were associated with substantially lower scores on immediate recall of the RCFT (Fig. 1). A similar, but weaker association was also seen with RCFT delayed recall. The only other tests that were significantly correlated with CSF analytes were MoCA and JoLO. The magnitude of the association with RCFT immediate recall was the strongest among all the cognitive measures. CSF biomarkers were inversely associated with RCFT immediate recall with the β coefficients per one SD increase of −0.91, −0.92, −0.79 and −0.79, respectively, for tTau, pTau, tTau:Aβ_42_ and pTau:Aβ_42_. Notably, no significant association was seen with any CSF analytes (or ratios) with measures of verbal episodic memory (Fig. 1 and **Supplemental Table S2**; AVLT).

### 3.3 Identification of CSF peptides associated with AD and stratification of risk among AsymAD cases

In previous work we identified changes in networks of brain-derived proteins in the CSF that discriminate between CN controls and patients with AD^20,34^. Multidimensional scaling analysis of a small set of CSF samples revealed differences that segregated CSF samples into AD-like and Control-like groups^34^. These results suggest that changes in specific proteins may allow stratification of AsymAD individuals into groups at higher or lower risk of transitioning to symptomatic AD. Using a targeted panel of 65 proteins that discriminate AD and Control CSF^21^, we applied machine learning-based feature selection algorithms to identify a set of peptides which distinguish CN/BM- controls from symptomatic AD cases. Levels of these peptides in AsymAD CSF were evaluated by a series of unsupervised and supervised learning algorithms to determine their proximity to CN/BM- controls or AD cases and to stratify AsymAD individuals into those who may be at lower or higher risk of progression to AD.

**Figure 2.**
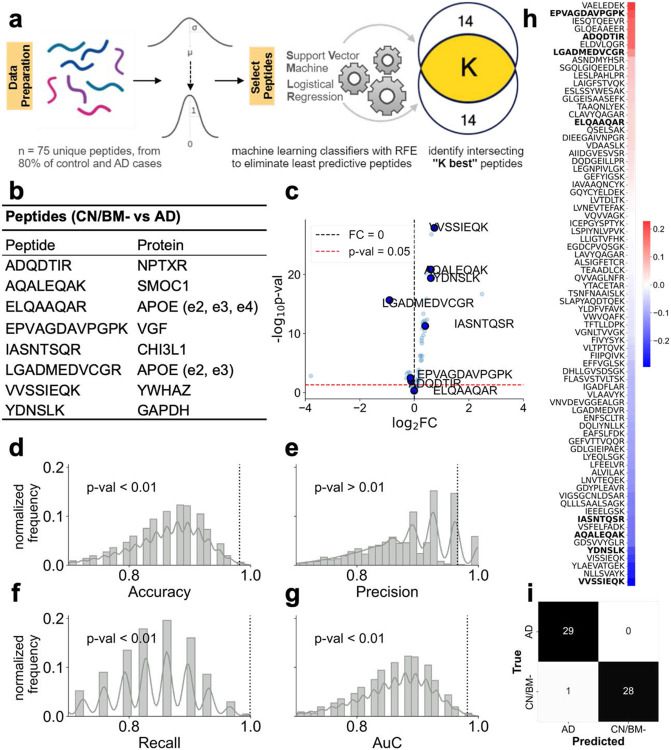
Selection and evaluation of peptides for classifying CN/BM- controls and symptomatic AD cases. (**a**) Schematic describing the process of peptide selection. The peptide selection procedure uses only the training dataset (80% of the controls and AD cases) and starts by mean centering and scaling the data to unit variance. Following this, RFE is run for peptide selection with two classifiers (SVM and logistic regression) in an independent fashion, which results in two sets of selected peptides. In each case, the RFE stopping criterion is set to 14 peptides. The peptides intersecting between these two sets are chosen in the final set. (**b**) The 8 peptides (and their associated proteins) which are chosen via the RFE approach. (**c**) A volcano plot showing log_2_FC (fold-change) vs −log_10_ p value. (**d-g**) Results from the random permutation test. 100000 random sets of 8 peptides are generated and their performance on classifying the validation data (20% held-out set) is evaluated using a logistic regression model. This is compared to the performance of the 8 peptides chosen via the RFE method (shown in the vertical dotted line). The metrices compared are accuracy, precision, recall, and ROC-AUC. For all scores except precision, the scores from the RFE derived peptides show p <0.01. (**h**) The Kendall’s-Tau correlation coefficient of the measured peptides with the MoCA score. When the peptides are sorted for the correlation coefficient, the 8 peptides in (**b**) are found to lie near the extremes indicating their stronger association with cognitive function. (**i**) Results from a logistic regression classifier on held-out data that played no role in peptide selection or model training. For 58 CN/BM- and AD subjects, it only misclassified one CN/BM- as AD.

Figure 2a shows a schematic for peptide biomarker selection using the machine learning strategy of Recursive Feature Elimination (RFE)^35^. The peptide biomarkers were identified by using RFE with two different linear classifiers (SVM and logistic regression), and then choosing only those peptides which appear in both selections. The training set for peptide selection used 80% of CN/BM- control and AD cases and the selected peptides were validated on the held-out 20% of the data. The selected peptides are shown in figure 2b. These peptides (n=8) distinguished CN/BM- from AD with over 98% classification accuracy (57 of 58 samples) on the held-out set using a logistic regression model^38^ (Fig. 2i). Such a high classification accuracy indicates that the chosen peptides are generalizable to unseen data and hence possess predictive value. These peptides also performed well on random permutation tests in which they were compared to randomly chosen sets of peptides for their classification ROC-AUC (Fig. 2d-g). Figure 2h shows Kendall-Tau correlation between all peptides measured across all subjects (CN/BM-, AsymAD, AD) and the MoCA score. The Kendall-Tau correlation shows the strength of monotonic association between the peptides and the MoCA score, and the coefficients are sorted in a decreasing order. The peptides that differentiate CN/BM- and AD cases (bolded in Fig. 2h) tend to appear on the extremes of the sorted correlation coefficients. These results suggest that the peptides chosen using the RFE approach classify CN/BM- and AD cases with very high accuracy and are also strongly associated to cognitive ability.

**Figure 3.**
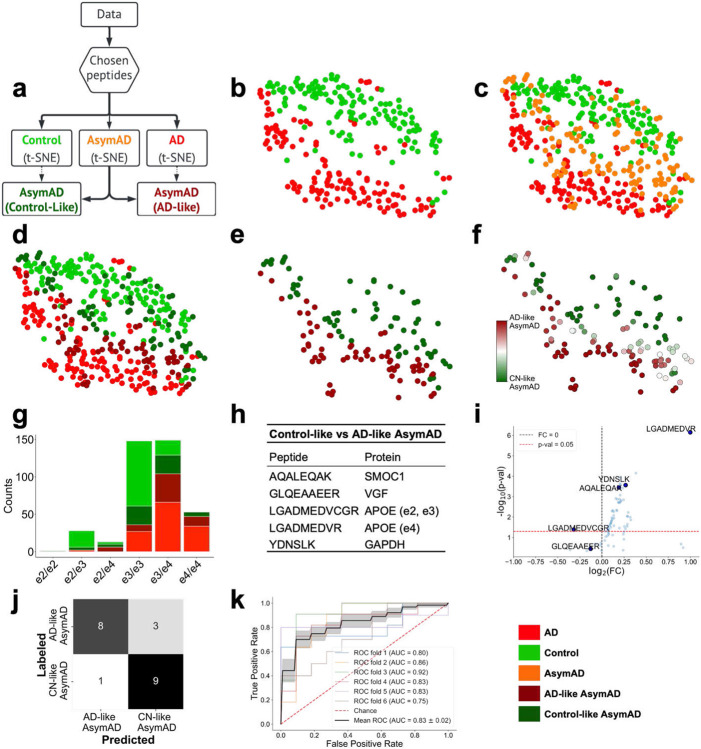
Sub-categorization of AsymAD cases and evaluation of JMI-selected peptides to classify subgroups. (**a**) Schematic showing the overview to split asymptomatic AD subjects into “Control-like” and “AD-like” sub-categories. These sub-categories are not present in the original data and are derived using an unsupervised methodology by computing proximity of AsymAD subjects to well-defined CN/BM- control and AD populations using the KNN (k=5) algorithm. (**b**) Two-dimensional representations of CN/BM- control and AD subjects using the t-SNE algorithm. These representations are computed using only the 8 peptides shown in figure 2b, which were predictive of the CN/BM- and AD groups. Hence, these low-dimensional representations are from two-levels of dimensionality reduction (peptide selection followed by t-SNE). A clear separation between the CN/BM- control and AD populations is noticeable. (**c**) AsymAD subjects overlaid with CN/BM- and AD subjects. AsymAD subjects extend between the CN/BM- control and AD subjects and do not fall on a distinct, separable region. This observation is used to sub-categorize AsymAD cases. (**d**) AsymAD subjects with greater proximity (computed using the KNN algorithm; k=5) to AD or CN/BM- control subjects are defined as AD-like or Control-like, respectively. (**e**) The stratified AsymAD cases (Control-like and AD-like) shown for clearer visualization. (**f**) The AsymAD stratification shown in (**b-e**) depends on t-SNE initialization. In order to study this sensitivity to t-SNE initialization, the steps (**b-e**) in the analysis are repeated 100 times, and the KNN (k=5) algorithm is used to stratify AsymAD individuals into Control-like or AD-like AsymAD as shown in (**d-e**). The color bar shows the probability of a subject being assigned Control-like (dark-green) or AD-like (maroon) subgroups. The middle whitish region in the color bar shows high uncertainty in AsymAD sub-category assignment. (**g**) Genotype profiles of the subjects in each category. The AD-like AsymAD cases (maroon) have a higher frequency of *APOE* ε4 allele, as compared to the Control-like AsymAD (dark green). This difference in ε4 allele frequencies is significant at the p<0.001 threshold using Fisher’s exact test. (**h**) Peptides that differentiate Control-like and AD-like AsymAD cases using the JMI criterion. An algorithm different from RFE was chosen to perform this step independently from the previous peptide identification step (between CN/BM- and AD cases). Only 80% of the AsymAD subjects were used to identify these peptides. (**i**) A volcano plot showing log_2_FC (fold-change) vs −log_10_ p value for the selected peptides in (**h**). (**j**) Confusion matrix showing classification performance on the 20% held-out AsymAD subjects. A logistic classifier was used on the 80% training AsymAD samples using peptides shown in (**h**). (**k**) ROC curves from 6-fold cross-validation to classify AsymAD subjects (into Control-like and AD-like AsymAD) using selected peptides shown in (**h**) with AUC between 0.75 – 0.92 with mean AUC of 0.83. The shaded region shows the standard error for mean ROC-AUC.

Figure 3 shows the low dimensional t-SNE analysis of the peptide data using the 8 RFE-selected peptides. Figure 3a shows the schematic of how AsymAD cases are sub-categorized into “Control-like” and “AD-like”, based on their proximity to CN/BM- controls and AD cases, respectively. Figure 3b-d shows 2-dimensional representation of the peptide data derived using t-SNE algorithm. While the CN/BM- and AD cases occur in separable clusters (Fig. 3b), the AsymAD cases extend between them (Fig. 3c). Such a result is expected given that these individuals are hypothesized to be in a transitional stage between CN/BM- controls and symptomatic AD. Figure 3d-e shows stratification of AsymAD into Control-like and AD-like groups by using a KNN (k=5) algorithm. AD-like AsymAD cases are those with ≥3 of 5 nearest neighbors among AD cases, and Control-like AsymAD cases are those with the majority of nearest neighbors among CN/BM- controls. The low dimensional t-SNE representations were computed from only those 8 peptide features which were chosen for distinguishing CN/BM- from AD cases. The sub-categories were compared for age, sex, race, education, cognitive performance, and levels of CSF Aβ_42_, tTau, and pTau (**Supplemental Fig. S1**). No significant difference was seen between the two AsymAD sub-categories for any of these features. In contrast, *APOE* profiles are significantly different (p=0.0011 by Fisher’s exact test) with higher ε4 allele frequency in the AD-like AsymAD cases (Fig. 3g), indicating a higher genetic risk for AD in these individuals.

We next applied Joint Mutual Information (JMI) algorithm to determine if a subset of peptides can be predictive of the Control-like and AD-like AsymAD sub-categories. Unlike RFE, JMI is an information theoretic approach free from the choice of a classifier. A set of 5 peptides was chosen by the JMI algorithm to stratify AsymAD sub-categories. Of these 5 peptides (Fig. 3h-i), two are directly linked to *APOE* genotype. LGADMEDVR is specific for the ApoE ε4 isoform, while LGADMEDVCGR is shared by ε2 and ε3 (muted for ε4/ε4 genotype). Note that 3 of the 5 peptides selected by the JMI algorithm to sub-categorize AsymAD cases were among the 8 peptides selected by RFE to discriminate between AD cases and CN/BM- controls (Fig. 2b). The ApoE peptide shared by ε2 and ε3 isoforms was also among those selected by RFE for discriminating AD cases and CN/BM- controls but the JMI algorithm selected a new peptide specific for ApoE ε4 for stratifying AsymAD cases. This result further supports an associative link between AsymAD sub-categories and *APOE* genotype and contrasts with the lack of difference between AD-like and Control-like subgroups in demographic features, cognitive performance, or levels of CSF Aβ_42_, tTau, or pTau (**Supplemental Fig. S1**). GLQEAAEER is associated to the VGF protein but is distinct from the VGF peptide selected by RFE. AQALEQAK to the SMOC1 protein and YDSLK to the housekeeping protein GAPDH are the same peptides selected by RFE. Together, these 5 peptides show 81% success in classifying sub-categories (using a linear logistic regression model) on 20% held-out AsymAD cases which had no role in JMI peptide selection or classifier training (Fig. 3j).

### 3.4 Validation of CSF peptides in ADNI data

**Figure 4.**
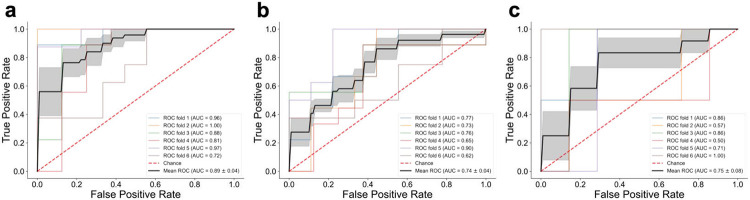
Evaluation of REF- and JMI-selected peptides in ADNI data. **(a)** Matched CN/BM- (n=52) and AD (n=52) individuals in the ADNI dataset using previously derived (n=8) peptides from the EHBS data (peptides shown in Fig. 2b) were classified with a mean ROC-AUC of 0.89. **(b)** Matched CN/BM- (n=52) and AsymAD (n=52) individuals in the ADNI data using (n=5) peptides shown in Fig. 3h were classified with a mean ROC-AUC of 0.74. **(c)** AsymAD with positive and negative FDG PET using (n=5) peptides shown in Fig. 3h were classified with a mean ROC-AUC of 0.75. In all cases, a 6-fold cross-validation approach is used with a linear logistic regression model. The shaded region shows the standard error for mean ROC-AUC.

In order to assess the sets of peptides selected by RFE for discriminating AD from CN/BM- controls and by JMI to stratify AD-like and Control-like AsymAD cases, we sought independent validation using available data from the ADNI cohort. Amyloid (AV45) PET results were used to determine the presence or absence of AD pathology. AV45 SUVR cutoff (>1.226) was determined based on ROC analysis of results from individuals classified as CN or Dementia at their baseline ADNI visit. Figure 4a shows the performance of (n=8) peptides which were previously identified for discriminating CN/BM- and AD cases in the EHBS dataset (Fig. 2b). In the ADNI dataset, when these peptides were used to classify CN/BM- controls (AV45 SUVR <1.226) and individuals with symptomatic AD (MCI or Dementia with AV45 SUVR >1.226), a 6-fold cross-validation approach using a linear logistic regression model gave a mean ROC-AUC of 0.89. For AsymAD cases (CN with AV45 SUVR>1.226), 52 cases were identified in ADNI. To test the ability of JMI-selected peptides (n=5; Fig. 3h) to stratify cognitively normal individuals, we used these peptides to classify the 52 ADNI AsymAD individuals with a demographically matched set of 52 CN/BM- (AV45 SUVR ≤1.226) individuals. The 5 JMI-selected peptides were able to classify these two groups with a mean AUC of 0.75 (Fig. 4b). Lastly, we took advantage of baseline FDG PET results to identify individuals with hypometabolism as a means of stratifying ADNI AsymAD individuals who might be closer to developing clinical symptoms. As was done with AV45 results, FDG PET SUVR cutoff (<1.191) was determined based on ROC analysis of results from individuals classified as CN or Dementia at their baseline ADNI visit. This cutoff identified 10 AsymAD cases with evidence of hypometabolism and 42 with normal FDG PET scans. Figure 4c shows the performance of (n=5) peptides previously identified by JMI algorithm for discriminating Control-like vs AD-like AsymAD cases in the EHBS cohort (Fig. 3). Despite small sample sizes, the mean ROC-AUC (6-fold cross-validation with a linear logistic regression model) for the ADNI dataset was 0.75 (0.81 with EHBS data). As in our sub-categorization of AsymAD cases in the EHBS cohort, demographic features (except gender), CSF AD biomarkers, and MoCA score were not different in the FDG PET-positive and -negative AsymAD subgroups (**Supplemental Fig. S2**). These results support the predictive ability of RFE- and JMI-selected peptide panels to discriminate CN/BM- controls from AD cases and to sub-categorize AsymAD cases, respectively, in an independent dataset.

## 4. Discussion

Our findings demonstrate that cognitively normal individuals with CSF biomarkers indicating underlying AD pathology (AsymAD) have distinct patterns of medical comorbidities, family history, neuropsychological measures, and CSF peptide levels compared to AD biomarker-negative controls. Machine learning approaches successfully stratified AsymAD cases to identify a sub-category whose CSF peptide profiles are more “AD-like” and another that is more “Control-like”. These results identify key features predicting progression to symptomatic AD that may serve to prioritize individuals for secondary prevention trials or for treatment with emerging disease-modifying therapies.

Numerous studies have established links between hypertension, diabetes, dyslipidemia, and peripheral artery disease and risk of AD and dementia^39,40^. In our study, we found evidence supporting a relationship between dyslipidemia and diabetes with AD biomarkers, specifically with CSF Aβ_42_ (but not Tau), among cognitively normal individuals (Table 2). We also found higher prevalence of dyslipidemia and diabetes as well as higher frequency of family history of dyslipidemia in biomarker-positive AsymAD individuals compared to a matched group of biomarker-negative controls (Table 1). Previous studies have shown increased levels of serum cholesterol in mid-life associated with increased risk of developing AD later in life^41-43^. In addition to its potent risk-modifying effect on AD^44^, ApoE plays a key role in cholesterol metabolism in the periphery, with the *APOE* ε4 allele associated with dyslipidemia and coronary heart disease^45-47^. In our study, *APOE* ε4 allele was enriched among individuals with AsymAD and was also a distinguishing feature between AD-like and Control-like AsymAD subgroups. Diabetes and metabolic syndrome have been strongly linked to AD risk^48^, including a strong association of mid-life diabetes with AD risk^49^. While hypertension in mid-life has also been reported to significantly increase the risk for late-life cognitive decline and AD^50,51^, results of epidemiological studies have been mixed^52^. In our study, we did not find significant differences in prevalence, family history, or relationship of hypertension to CSF AD biomarkers among cognitively normal individuals. As we stratified groups solely based on objective measures of CSF AD biomarkers, the relationships that we identified are not dependent on clinical classifications, and these findings support a relationship between dyslipidemia and diabetes with early evolution of AD pathology, specifically mediated through effects on Aβ_42_ during clinically silent asymptomatic stages.

In contrast to the association of medical comorbidities and family history with levels of CSF Aβ_42_, we found significant relationships between CSF tTau and pTau, but not Aβ_42_, with cognitive test results. In our comparison of 134 matched biomarker-positive and -negative groups in the EHBS cohort, only the score on a visuospatial task (Judgment of Line Orientation; JoLO) differed significantly between groups. It should be noted that since all individuals in the EHBS cohort are cognitively normal at enrollment, these comparisons are likely limited by a strong ceiling effect. When we examined the relationship of these scores to CSF AD biomarkers in the larger cohort (n=1149) with more robust statistical methods, we identified several additional relationships. Episodic memory, which is typically the earliest domain affected in AD^53^, was measured by two primary measures, the Rey Auditory Verbal Learning Test (AVLT) and the Rey-Osterreith Complex Figure Test (RCFT). Interestingly, visual memory (RCFT) but not verbal memory (AVLT) was associated with CSF levels of tTau and pTau (p<0.0001; Fig. 1), and the parameter estimates for RCFT immediate recall (−0.91 tTau and −0.92 pTau) were high. JoLO was not significantly correlated with individual analytes, but was significantly associated with the ratios of tTau and pTau to Aβ_42_ (Table 4). These findings suggest that visual memory and visuospatial abilities may be more sensitive to very early changes in AD than language-based abilities. While this is a fairly novel conclusion, there are some precedents supporting this possibility^54-56^, and our results highlight the importance of assessing visuospatial abilities to identify the earliest cognitive changes associated with AD.

The present study showed that a small set of 8 differentially expressed peptides can effectively distinguish AD cases from cognitively healthy controls. These peptides were identified using machine learning algorithms (Recursive Feature Elimination – RFE, combined with linear classifiers) and show good generalizability on unseen data which had no role in peptide identification. Importantly, the set of predictive peptides have been shown in recent studies to be important in tracking disease status and progression. Neuronal Pentraxin Receptor (NPTXR) isoform 1 (protein for the ADQDTIR peptide) has been shown to be a CSF biomarker of AD progression^57^ with levels differing between MCI and more advanced AD stages. YWHAZ (protein for the VVSSIEQK peptide) has recently emerged as an important biomarker to discriminate AD from non-AD cases with cognitive impairment and also predicts individuals with high Tau and low Aβ_42_ levels^58^. CHI3L1 (protein for the IASNTQSR peptide; also known as YKL-40) has been reported in other studies as a potential prognostic fluid biomarker, and its ratio to Aβ_42_ is predictive for developing cognitive impairment^59^. CHI3L1 is also a glial/inflammation related biomarker^17,58,60^. VGF (protein for the EPVAGDAVPGPK peptide) has been strongly associated with cognitive trajectory and suggested to act through mechanisms independent of amyloid plaques and neurofibrillary tangles in contributing to cognitive decline^61^. Further, VGF has also been identified as a key regulator playing a causal role in protecting against AD pathogenesis and progression^62^. SMOC1 (protein for AQALEQAK peptide), which is related to the extracellular matrix and strongly correlated with global AD pathology in brain^63^, has shown the ability to discriminate between AD and non-AD cognitive impairment (specificity for AD) and to predict levels of CSF Aβ_42_, tTau, and pTau^58^. GAPDH (protein for the YDNSLK peptide) is known to form stable aggregates with extracellular Aβ, and these aggregates have been found to be proportional to the progressive stage of AD^64,65^. These peptides from 8 proteins, each with plausible biological connection to AD pathophysiology, were found to be among the most strongly associated with cognition and were able to discriminate CSF samples from AD patients and Controls with 98% accuracy (Fig. 2).

The 15-20 year period during which AD neuropathology evolves silently prior to cognitive decline offers a window of opportunity to slow or prevent clinical disease. However, as many individuals with AD neuropathology never develop symptoms during life, it will be critical that we develop tools to identify those individuals at greater risk of cognitive decline. Toward this goal, we applied a stratification strategy to sub-categorize 134 cognitively normal individuals with asymptomatic AD into AD-like and Control-like groups using expression levels of the 8 peptides which were selected for differentiating AD cases from healthy, biomarker-negative controls. The AD-like AsymAD cases show a higher frequency of the *APOE* ε4 allele but are otherwise indistinguishable from the Control-like AsymAD cases based on demographics, cognitive performance, or level of CSF Aβ_42_, tTau, or pTau. Applying a Joint Mutual Information (JMI) algorithm, we identified an independent set of 5 peptides (from SMOC1, VGF, APOE ε2/ε3, APOE ε4, and GAPDH) which were able to classify AD-like and Control-like AsymAD cases with 81% accuracy. The two ApoE-associated peptides selected by JMI specifically discriminate presence or absence of the ε4 isofom. While there have been conflicting reports regarding the impact of *APOE* genotype on clinical progression among symptomatic AD patients^66-70^, the selection of these peptides and higher frequency of *APOE* ε4 in the AD-like AsymAD subgroup suggests that ε4 carriers are predisposed to more rapid decline and a shortened asymptomatic phase of AD.

To test the peptides identified in the EHBS cohort, we took advantage of targeted proteomics analysis which our group recently performed on 706 baseline ADNI CSF samples. Since amyloid PET scans were available for the ADNI cohort, we used AV45 PET positivity as a means of defining individuals with underlying AD pathology. The 8 RFE-selected peptides were effective in discriminating between CN/BM- (AV45 PET negative) controls and amyloid PET-confirmed AD with mean AUC of 0.89. Among all CN individuals in ADNI, there were 52 with asymptomatic AD based on positive amyloid PET. The 5 JMI-selected peptides were able to discriminate these AsymAD cases from a demographically-match cohort of 52 CN individuals with negative AV45 PET scans with a mean AUC of 0.74. Only a very small number of individuals in ADNI have transitioned from CN to MCI or Dementia during longitudinal follow up, and, in addition to being a rare event, clinical progression is complicated by frequent reversions from MCI to CN^71-73^. To avoid these limitations, we used FDG PET to identify AsymAD individuals with evidence of hypometabolism and presumably at greater risk of symptomatic progression. Despite small sample size (n=10), the 5 JMI-selected peptides classified FDG-positive AsymAD cases with mean AUC of 0.75. Unlike our previous studies with deep proteomics and network analyses^20,34^, the purpose of the current work was to evaluate CSF peptides that might serve as effective biomarkers to predict cognitive decline in cognitively normal individuals harboring AD pathology. Deep proteomics comparing AD-like and Control-like AsymAD cases should produce better understanding of changes occurring during the transition from asymptomatic to symptomatic stages of AD, and additional work will refine peptide panels to improve their predictive ability. Longitudinal follow up of individuals with asymptomatic AD will be required for ultimate validation of predictive biomarkers, and this will be possible in the EHBS cohort over time.

In sum, our current findings provide evidence linking dyslipidemia and diabetes (but not hypertension) to early evolution of AD pathology mediated through effects on Aβ_42_ in healthy middle-aged individuals. On cognitive measures, we identified a strong relationship of CSF tTau and pTau levels (but not Aβ_42_) with visual episodic memory and visuospatial perception, which was not observed with language-based tests, suggesting earlier impact on visual networks in the evolution of AD pathology. Lastly, using machine-learning algorithms we identified peptide panels that can stratify individuals at greater risk of progression to symptomatic disease who can be prioritized for prevention trials and for treatment with emerging disease-modifying therapies for AD.

## Figures and Tables

**Table 1:** Patients Characteristics, Medical History and Family History

Characteristics	Clinical AD	AsymAD	CN/BM-	p value
Age, mean±SD	66.0±5.8	66.0±5.8	65.9±6.0	0.11
Female, n (%)	100 (75)	100 (75)	100 (76)	0.99
Race, n (%)				0.99
Caucasian	124 (92.5)	124 (92.5)	124 (92.5)	
African American	9 (6.7)	9 (6.7)	9 (6.7)	
Asian	1 (0.7)	1 (0.7)	1 (0.7)	
Education, mean±SD	15.4±2.6	16.7±2.0	16.8±2.3	0.69
MoCA, mean±SD	17.4±5.5	26.3±2.6	26.8±2.0	0.07
*APOE* ε4 Allele Frequency	0.50	0.40	0.08	**<0.0001**
CSF Analytes, median (IQR)				
Aβ_42_ pg/ml	540.7 (445.6-660.2)	740.1 (609.8-862.5)	1412.0 (1192-1700)	**<0.0001**
tTau pg/ml	343.2 (265.7-458.5)	242.0 (194.9-299.4)	167.6 (139.9-192.7)	**<0.0001**
pTau pg/ml	33.9 (26.7-47.3)	22.8 (18.6-28.2)	14.8 (12.2-17.2)	**<0.0001**
tTau:Aβ_42_ ratio	0.64 (0.49-0.86)	0.31 (0.27-0.42)	0.12 (0.11-0.13)	**<0.0001**
pTau:Aβ_42_ ratio	0.065 (0.047-0.088)	0.029 (0.025-0.042)	0.011 (0.002-0.011)	**<0.0001**
**Medical History**				
Hypertension, n (%)		51 (38.1)	38 (28.4)	0.11
Diabetes, n (%)		17 (12.7)	6 (4.5)	**0.033**
Dyslipidemia, n (%)		84 (62.7)	65 (48.5)	**0.015**
CAD, n (%)		7 (5.2)	2 (1.5)	0.10
**Family History**				
MCI, n (%)		47 (35.1)	35 (26.1)	0.30
AD, n (%)		49 (36.6)	20 (14.9)	**0.0004**
Memory Loss, n (%)		76 (56.7)	64 (47.8)	0.52
Hypertension, n (%)		99 (73.9)	90 (67.2)	0.43
Diabetes, n (%)		50 (37.3)	39 (29.1)	0.45
Dyslipidemia, n (%)		86 (64.2)	74 (55.2)	**0.030**
CAD, n (%)		69 (51.5)	76 (56.7)	0.55
Stroke, n (%)		47 (35.1)	48 (35.8)	0.41

The table shows demographic features, CSF analytes levels, medical history, and family history for symptomatic AD, AsymAD (CSF biomarker positive) and CN/BM- (CSF biomarker negative) groups. The AsymAD cases and CN/BM- controls are matched for age, sex, race, and education. P values are for comparisons of AsymAD and CN/BM- groups. Continuous variables were compared by paired t-test or Wilcoxon signed rank test depending on the distribution. Categorical variables were compared with McNemar-Bowker’s test.

**Table 2.** Summary of Correlation of CSF Analytes with Medical and Family History

Medical/Family History	CSF Analyte	Parameter estimate	P value	FDR<20%
Dyslipidemia	Standardized Aβ42	**−0.23 (−0.40, −0.07)**	**0.005**	**YES**
Dyslipidemia	Standardized tTau	0.04 (−0.09, 0.18)	0.543	-
Dyslipidemia	Standardized pTau	0.04 (−0.10, 0.17)	0.605	-
Dyslipidemia	Standardized tTau:Aβ_42_	**0.07 (0.02, 0.12)**	**0.004**	**YES**
Dyslipidemia	Standardized pTau:Aβ_42_	**0.04 (0.00, 0.08)**	**0.045**	-
Diabetes	Standardized Aβ_42_	**−0.42 (−0.78, −0.06)**	**0.021**	-
Diabetes	Standardized tTau	0.11 (−0.08, 0.30)	0.270	-
Diabetes	Standardized pTau	−0.01 (−0.27, 0.24)	0.927	-
Diabetes	Standardized tTau:Aβ_42_	**0.19 (0.08, 0.30)**	**0.001**	**YES**
Diabetes	Standardized pTau:Aβ_42_	**0.10 (0.02, 0.18)**	**0.010**	-
Family history AD	Standardized Aβ_42_	**−0.33 (−0.54, −0.12)**	**0.002**	**YES**
Family history AD	Standardized tTau	**0.15 (0.01, 0.29)**	**0.040**	-
Family history AD	Standardized pTau	0.12 (−0.03, 0.28)	0.113	-
Family history AD	Standardized tTau:Aβ_42_	**0.16 (0.08, 0.22)**	**<0.0001**	**YES**
Family history AD	Standardized pTau:Aβ_42_	**0.08 (0.02, 0.14)**	**0.007**	-

Summary of median regression analysis showing significant effects of medical and family history on CSF biomarkers. History of dyslipidemia, diabetes, and family history of AD compared with individuals negative for these features had significant effects on CSF analytes. Associations with Aβ_42_ and tTau:Aβ_42_ ratio remain significant after FDR correction (only tTau:Aβ_42_ ratio with diabetes). No association was seen with tTau or pTau levels. Results reported as standardized parameter estimates with 95% confidence intervals.

**Table 3.** Comparison of Cognitive Performance between CSF Biomarker Positive and Negative Groups

Cognitive Measure	AsymAD (n=134)	CN/BM- (n=134)	P value
MoCA total score	27.0 (25.0 - 28.0)	27.0 (25.0 - 28.0)	0.12
Rey AVLT			
Trial A5	11.0 (10.0 - 13.0)	12.0 (10.0 - 13.5)	0.79
Immediate recall	8.0 (7.0 - 12.0)	10.0 (6.5 - 12.0)	0.60
Delayed recall	5.0 (4.0 - 7.0)	6.0 (4.0 - 7.0)	0.82
Animal Fluency	21.0 (19.0 - 25.0)	22.0 (19.5 - 25.0)	0.83
Phonemic Fluency	45.0 (38.0 - 52.0)	43.0 (36.0 - 51.0)	0.17
MINT	31.0 (30.0 - 32.0)	32.0 (30.0 - 32.0)	0.40
Number Span Forward	7.0 (6.0 - 8.0)	7.0 (6.0 - 8.0)	0.76
Number Span Backward	5.0 (4.0 - 6.0)	5.0 (5.0 - 6.0)	0.83
Trails A, seconds	33.0 (27.0 - 43.0)	31.5 (26.0 - 39.0)	0.23
Trails B, seconds	67.8 (56.0 - 84.0)	68.0 (56.0 - 82.0)	0.79
RCFT			
Copy	33.0 (30.0 - 34.0)	33.0 (30.5 - 35.0)	0.38
Immediate recall	16.5 (12.0 - 20.5)	16.5 (13.5 - 22.3)	0.12
Delayed recall	15.5 (10.5 - 20.0)	16.5 (12.3 - 21.0)	*0.09*
JoLO, total score	25.0 (23.0 - 28.0)	26.0 (24.5 - 28.0)	**0.0022**

Neuropsychological test scores are reported for the AsymAD and biomarker-negative control groups. Values are median (interquartile range). Comparisons show that for most tests, there is no difference between group. However, AsymAD patients performed significantly worse in Judgment of Line Orientation (JoLO; p=0.0022), and tended to recall fewer items in RCFT delayed recall (p=0.09). All p values are computed using the Wilcoxon signed rank test.

**Table 4.** Summary of Significant Correlations of CSF Biomarkers with Cognitive Measures

Cognitive Measure	CSF Analyte	Parameter estimate	P value	FDR<20%
MoCA	Standardized Aβ_42_	0.06 (−0.10, 0.22)	0.470	-
MoCA	Standardized tTau	**−0.22 (−0.37, −0.07)**	**0.005**	**YES**
MoCA	Standardized pTau	**−0.23 (−0.37, −0.09)**	**0.002**	**YES**
MoCA	Standardized tTau:Aβ_42_	**−0.26 (−0.41, −0.11)**	**0.001**	**YES**
MoCA	Standardized pTau:Aβ_42_	**−0.25 (−0.41, −0.09)**	**0.002**	**YES**
RCFT Immediate	Standardized Aβ_42_	−0.40 (−0.95, 0.15)	0.150	-
RCFT Immediate	Standardized tTau	**−0.91 (−1.30, −0.53)**	**<0.0001**	**YES**
RCFT Immediate	Standardized pTau	**−0.92 (−1.37, −0.47)**	**<0.0001**	**YES**
RCFT Immediate	Standardized tTau:Aβ_42_	**−0.79 (−1.30, −0.27)**	**0.003**	**YES**
RCFT Immediate	Standardized pTau:Aβ_42_	**−0.79 (−1.26, −0.31)**	**0.001**	**YES**
RCFT Delayed	Standardized Aβ_42_	0.05 (−0.39, 0.49)	0.810	-
RCFT Delayed	Standardized tTau	−0.52 (−1.06, 0.02)	0.060	-
RCFT Delayed	Standardized pTau	**−0.62 (−1.15, −0.09)**	**0.022**	-
RCFT Delayed	Standardized tTau:Aβ_42_	−0.68 (−1.44, 0.09)	0.080	-
RCFT Delayed	Standardized pTau:Aβ_42_	−0.72 (−1.55, 0.10)	0.090	-
JoLO	Standardized Aβ_42_	0.18 (−0.02, 0.39)	0.080	-
JoLO	Standardized tTau	−0.13 (−0.37, 0.10)	0.260	-
JoLO	Standardized pTau	−0.13 (−0.37, 0.12)	0.320	-
JoLO	Standardized tTau:Aβ_42_	**−0.29 (−0.55, −0.04)**	**0.025**	-
JoLO	Standardized pTau:Aβ42	**−0.37 (−0.61, −0.12)**	**0.003**	**YES**

Summary of median regression analysis showing significant effects of CSF biomarkers on cognitive test performance. Increases in tTau, pTau, and ratio of tTau and pTau to Aβ_42_ were associated with poorer scores on MoCA, RCFT immediate and delayed recall, and judgment of line orientation (JoLO). Strongest associations were seen with performance on RCFT immediate recall. Associations remain significant after correction for multiple corrections with the exception of RCFT delayed recall. Levels of Aβ_42_ were not significantly associated with any cognitive tests. Results reported as standardized parameter estimates with 95% confidence intervals.
